# Recent increase in low complexity polygenomic infections and sialic acid-independent invasion pathways in *Plasmodium falciparum* from Western Gambia

**DOI:** 10.1186/s13071-023-05929-4

**Published:** 2023-08-31

**Authors:** Nora Nghochuzie Nganyewo, Fatoumata Bojang, Eniyou Cheryll Oriero, Ndey Fatou Drammeh, Olumide Ajibola, Haddijatou Mbye, Aminata Seedy Jawara, Simon Corea, Gordon Akanzuwine Awandare, Umberto D’Alessandro, Lucas N. Amenga-Etego, Alfred Amambua-Ngwa

**Affiliations:** 1 Medical Research Council Unit The Gambia at London, School of Hygiene and Tropical Medicine, Banjul, The Gambia; 2 West African Centre for Cell Biology of Infectious Pathogens (WACCBIP), University of Ghana, Accra, Ghana

**Keywords:** Erythrocyte, Invasion, *P. falciparum*, Ligand genes, Expression, Malaria, Inhibition

## Abstract

**Background:**

The malaria parasite *Plasmodium falciparum* utilizes multiple alternative receptor-ligand interactions for the invasion of human erythrocytes. While some *P. falciparum* clones make use of sialic acid (SA) residues on the surface of the human glycophorin receptors to invade the erythrocyte, others use alternative receptors independent of sialic acid residues. We hypothesized that over the years, intensified malaria control interventions and declining prevalence in The Gambia have resulted in a selection of parasites with a dominant invasion pathways and ligand expression profiles.

**Methods:**

Blood samples were collected from 65 malaria-infected participants with uncomplicated malaria across 3 years (2015, 2016, and 2021). Genetic diversity was determined by genotyping the merozoite surface protein 2 (*msp2*) polymorphic gene of *P. falciparum.* Erythrocyte invasion phenotypes were determined using neuraminidase, trypsin, and chymotrypsin enzymes, known to cleave different receptors from the surface of the erythrocyte. Schizont-stage transcript levels were obtained for a panel of 6 *P. falciparum* invasion ligand genes (*eba175, eba181, Rh2b, Rh4, Rh5*, and *clag2*) using 48 successfully cultured isolates.

**Results:**

Though the allelic heterozygosity of *msp2* repeat region decreased as expected with reduced transmission, there was an increase in infections with more than a single *msp2* allelotype from 2015 to 2021. The invasion phenotypes of these isolates were mostly SA independent with a continuous increase from 2015 to 2021. Isolates from 2021 were highly inhibited by chymotrypsin treatment compared to isolates from 2015 and 2016. Higher invasion inhibition for 2021 isolates was further obtained following erythrocyte treatment with a combination of chymotrypsin and trypsin. The transcript levels of invasion ligand genes varied across years. However, levels of *clag2*, a rhoptry-associated protein, were higher in 2015 and 2016 isolates than in 2021 isolates, while *Rh5* levels were higher in 2021 compared to other years.

**Conclusions:**

Overall, these findings suggest increasing mixed infections with an increase in the use of sialic-acid independent invasion pathways by *P. falciparum* clinical isolates in the Western part of Gambia.

**Graphical Abstract:**

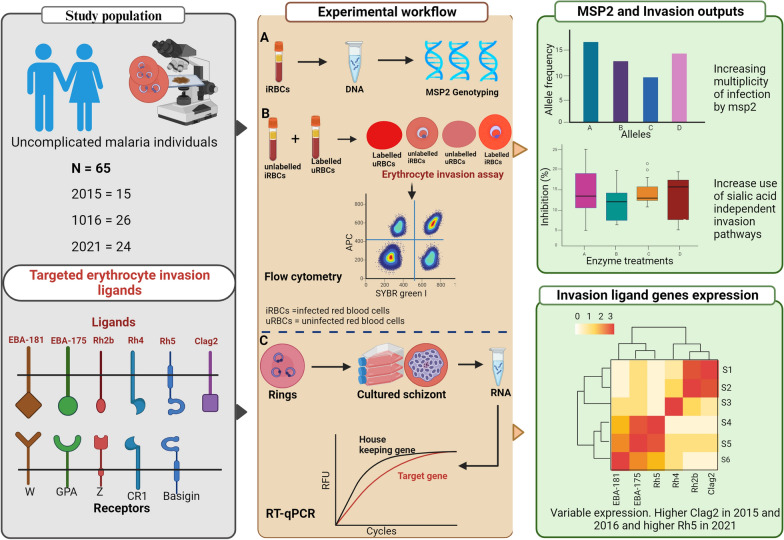

**Supplementary Information:**

The online version contains supplementary material available at 10.1186/s13071-023-05929-4.

## Background

*Plasmodium falciparum* malaria parasite invade and multiply in human red blood cells (RBCs) during the entire erythrocytic phase of its life cycle. Erythrocyte invasion by *P. falciparum* merozoite stages is a crucial, complex, and multistep process requiring multiple alternative receptor-ligand interactions [1]. *Plasmodium falciparum* ligands are made up of two protein families: the erythrocyte binding antigen (EBA-175, EBA-140, EBA-165, EBL1, and EBA-181) [2–5] and the reticulocyte binding protein-like homologs (Rh1, RH2A, Rh2B, Rh4, and Rh5) [6–9]. These ligands determine *P. falciparum* invasion phenotypes, which have mostly been characterized by the treatment of erythrocytes with enzymes known to cleave part of the receptor repertoire on which they bind. Commonly used enzymes include neuraminidase, which cleaves sialic acids from the glycophorin (Gly) receptors (GlyA, B, and C), trypsin cleaves glycophorins A, C, and complement receptor 1 (CR1), while chymotrypsin cleaves GlyB, band 3, and other invasion-related proteins [10, 11]. Thus, based on the sialic acid (SA) residues of the glycophorin receptors, *P. falciparum* invasion pathways are described as either SA dependent or SA independent. These ligand-receptor interactions are variably used by *P. falciparum* isolates in different malaria populations, and some parasite lines activate alternative pathways or switch invasion phenotypes during the in vitro life cycle [12–15].

Previous studies in The Gambia have reported the predominant use of the SA-dependent pathways during a period of relatively higher malaria transmission [16, 17]. Over the last 20 years, malaria transmission has been drastically reduced because of intensified and sustained malaria control interventions. Currently, The Gambia is moving towards malaria elimination (pre-elimination phase) with an overall prevalence of 0.2% in the country and incidence of 25–100 cases per 1000 population in the Western region [18]. Hence, how this transition from high to low malaria transmission has affected erythrocyte invasion mechanisms, which involves ligands targeted for vaccine development, is not known.

In this study, we investigated the invasion phenotypes and transcript levels of six invasion ligand genes (*eba175, eba181, Rh2b, Rh4, Rh5, clag2*) of *P. falciparum* field isolates from three different years in The Gambia (2015, 2016, and 2021). We also used the highly polymorphic *P. falciparum* surface protein, merozoite surface protein 2 (*msp2*), to assess the complexity of infections from these different years. We hypothesize that intensified malaria control interventions and declining prevalence in The Gambia have resulted in a selection of parasites with the most dominant invasion pathways driven by expression of specific ligands.

## Methods

### Study site and malaria sampling

This study was conducted in the Western Region of The Gambia. Uncomplicated malaria patients attending two health facilities (Brikama and Fajikunda health centres) who tested positive for *P. falciparum* by immunochromatic rapid diagnostic testing (RDT) and had reported not taking antimalarial drugs during the preceding 3 days were invited to participate in the study.

Patients were recruited in 2015 (*n* = 15), 2016 (*n* = 26), and 2021 (*n* = 24) during the annual peak of malaria transmission (September to December). Isolates from 2015 and 2016 were collected from children ≤ 14 years old while those from 2021 were collected from all age groups. The inclusion criteria were patients with axillary temperatures of > 37.5 °C, or a history of fever in the previous 48 h, and a positive RDT test for malaria. For each consenting qualified case, approximately 2 ml venous blood was collected into EDTA anticoagulant tubes and further confirmed for malaria by light microscopy. Plasma was removed and infected erythrocytes separated from lymphocytes and buffy coat by NycoPrep gradient centrifugation. The infected erythrocytes of samples collected in 2015 were cryopreserved in glycerolyte and stored in liquid nitrogen until use while those collected in 2016 and 2021 were directly resuspended at 2% hematocrit in Roswell Park Memorial Institute 1640 complete medium (RPMI 1640) (incorporating 25 mmol/l HEPES, 2 mmol/l, l glutamine, 25 mmol/l glucose, 25 mg of Gentamicin per litre, 10 mg of hypoxanthine per litre, and 10% human AB serum or 5000 mg/l of Albumax) and used for invasion assays.

### DNA extraction and *msp2* genotyping

For all patients included in the study, blood was spotted on filter papers (Whatman 3 mm, GE Healthcare, USA) prior to culture (day zero samples), labelled, air-dried, and stored in sealed plastic bags at 4 °C. *Plasmodium falciparum* DNA was subsequently extracted using the QIAamp DNA Mini Kit (Qiagen, Germany) according to the Qiagen-DNA purification from dried blood spot protocol. Merozoite surface protein 2 (*msp2*) repeat genotyping was done using specific primers as previously described [19]. Briefly, the primary PCR targeted the polymorphic block 3 region of *msp2* while the nested PCR primer sets were specific to *msp2* allelic families (3D7 and FC27). The primary PCR was carried out in a final volume of 15 μl containing 2 μl gDNA, 0.076 U/μl Taq polymerase (New England Biolabs), 250 nM forward and reverse primers, 125 μM dNTPs, 1× (1.5 μl) Thermopol buffer (New England Biolabs), and 10.35 μl nuclease-free water. Nested PCR was done at a final volume of 20 μl containing 1 μl of the primary PCR product, 0.05 U/μl Taq polymerase (New England Biolabs), 125 nM forward and reverse primers, 100 μM dNTPs, 1× (2 μl) Thermopol buffer (New England Biolabs) and 16.1 μl nuclease-free water. Cycling conditions used were as previously described [20] with positive (3D7 and Dd2) and negative (nuclease-free water) controls incorporated in each PCR run. Amplificons from the allelic families were analysed using QIAxcel (Qiagen) and fragment sizes were determined using QIAxcel ScreenGel Software.

### *Plasmodium falciparum* ligand gene expression assays

Isolates from samples with adequate volumes and at least 0.5% parasitaemia were cultured to the schizont stage and frozen at − 80 °C in three parts of TRIzol reagent (Ambion/Life Technologies, USA) as previously described [17]. RNA was extracted with the phenol-chloroform RNA extraction protocol according to the instructions of the Tri Reagent® manufacturer and concentrations were estimated using the Qubit fluorometer (Life Technologies, UK). RNA from each isolate was reverse transcribed using the First Strand cDNA Synthesis Kit (New England Biolabs) and the transcript levels for *eba175, eba181, clag2, Rh2b, Rh4*, and *Rh5* genes were determined using gene-specific primer/probe sets [17, 21, 22]. Reverse transcriptase quantitative PCRs were performed using the TaqMan universal PCR Master Mix (ThermoFisher) in 15 μl volumes with 330 nM concentrations of each primer and 160 nM concentrations of each probe at 95 °C for 10 min followed by 40 cycles of 95 °C for 30 s and 50 °C for 1:30 s. Each run included controls and 3D7 genomic DNA standards, with standard curves generated for each run. The threshold fluorescence value for each run was determined automatically by the BioRad CFX96 software.

### Erythrocyte invasion assays

Erythrocytes used for invasion assays were from blood group O^+^ uninfected human volunteers who had not taken antimalarial in the previous 1 month and were not on any medication. Fresh target erythrocytes were treated weekly with neuraminidase (Sigma-Aldrich, UK), trypsin (Sigma-Aldrich, UK), and chymotrypsin (Sigma-Aldrich, UK) in different combinations and concentrations as previously described [23] with some modifications. Briefly, the target erythrocytes were treated with 66.7 mU/ml of neuraminidase (NM), 66.7 µg/ml for low trypsin (LT), 1.0 mg/ml for high trypsin (HT); chymotrypsin and low trypsin (CHY_LT) with 1.0 mg/ml and 66.7 µg/ml, respectively, and 1.0 mg/ml chymotrypsin (CHY). Complete RPMI media were used as the negative control (no invasion inhibition) and a combination of all enzymes was used as the positive control (100% inhibition). All enzyme treatments of erythrocytes were performed at 37 °C for 1 h, followed by two washes with 1× PBS and once with RPMI 1640, and suspension at 2% hematocrit. The treated erythrocytes at 2% hematocrit were fluorescently labelled with Cell Trace^(R)^ Far Red (CTFR) Proliferation Kit (Invitrogen, C34564) as previously described [17, 23].

The invasion phenotypes of *P. falciparum* clinical isolates across 3 years, 2015 (*n* = 15), 2016 (*n* = 26), and 2021 (*n* = 24) were determined through their ability to invade receptor-depleted CTFR-labelled target erythrocytes. Assays were set up in triplicate in flat-bottom 96-well plates. For each assay, ring-stage parasite isolates from infected patients were added to CTFR-stained erythrocytes in a 1:1 ratio at 2% hematocrit in flat-bottomed 96-well culture plates for a total culture volume of 100 µl per well. Each isolate was tested in triplicate per enzyme-treated cell and plates were incubated at 37 °C in a gas atmosphere of 1% O_2_, 3% CO_2_, and 96% N_2_. After the first complete invasion cycle (48 h), cells were labelled with SYBR Green I nucleic acid gel stain (Invitrogen) to differentiate infected from uninfected erythrocytes. Invasion levels were determined using BD Accuri™ C6 Plus flow cytometer (BD Biosciences), and the percentage of cells stained with both CTFR and SYBR Green I was recorded as newly re-invaded.

### Statistical analyses

For *msp2* genotyping, the multiplicity of infection (MOI) was calculated by dividing the total number of alleles detected by the total number of samples [24] while the expected heterozygosity (a measure of genetic diversity) was used to assess population structure of parasites. Heterozygosity (He) was calculated using the formula

$${\text{He}}\, = \,\left[ {n/\left( {n - {1}} \right)} \right]\left[ {\left( {{1} - \Sigma {\text{Pi2}}} \right)} \right],$$

where *n* is the sample size and Pi is the allele frequency as previously described [25].

Gene transcript expression and invasion data analyses were performed with R version 4.2.0 software. The Gaussian distribution of data was assessed by the Shapiro-Wilk normality test, and variables that passed the test for normality were analysed using parametric methods; otherwise, non-parametric procedures were used. Enzyme inhibition rates and relative gene expressions were compared across sampling years using the Kruskal-Wallis test, and post hoc pairwise comparisons were performed using the Mann-Whitney *U* test with Bonferroni correction of multiple pairwise comparisons. Correlations between the distributions of the different enzyme inhibition phenotypes were tested by Spearman’s rho. *P* values < 0.05 were considered statistically significant for all analyses.

## Results

### Participant’s characteristics

A total of 65 malaria-infected people were retained with the lowest proportion from 2015 (23.1%) and similar proportions from 2016 and 2021 (40% and 36.9%, respectively). While the mean age was similar between the 2015 and 2016 participants (9 and 8.21, respectively), it was higher for 2021 participants (20.48). The proportion of male-to-female participants in the study was not different (31 and 30, respectively), and four participants did not indicate their gender. Samples collected in 2015 had higher parasitaemia ranging from 1 to 7% with a median of 1.85% compared to 2016 and 2021 with parasitaemia ranging from 0.21 to 5.2% (median 1.24%) and 0.4 to 3.6% (median 1%), respectively (Table 1).

**Table 1:** Characteristics of study participants from Western Gambia across three different years

Variables	2015	2016	2021
Number (%)	15 (23.07)	26 (40)	24 (36.92)
Mean age ± SD	9 ± 3.74	8.21 ± 3.44	20.48 ± 10.28
Male	8 (61.5)	10 (41.7)	13 (54.2)
Female	5 (38.5)	14 (58.3)	11 (45.8)
Median parasitaemia (range)	1.85 (1–7%)	1.24 (0.21–5.2%)	1(0.4–3.6%)

*SD* standard deviation

### Genetic diversity of *P. falciparum* infections

**Fig. 1:**
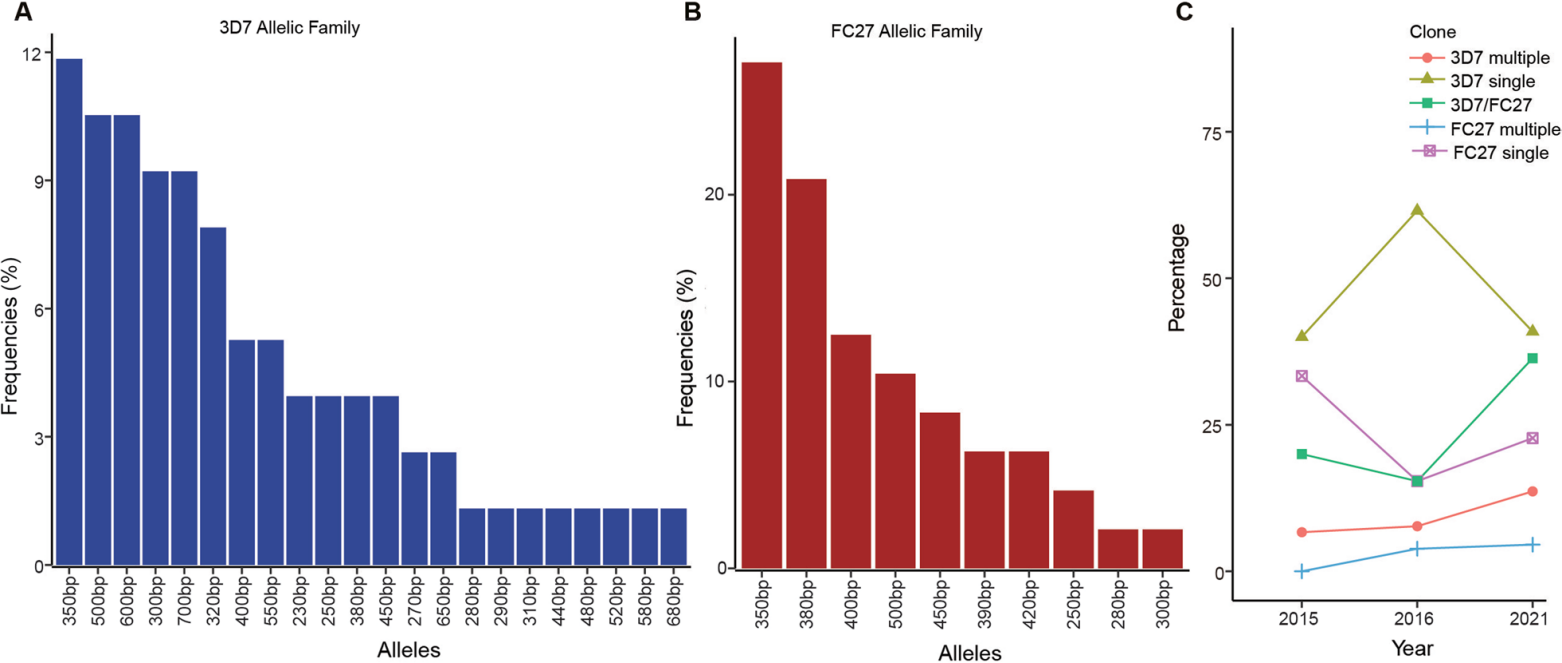
Prevalence and allelic frequencies of *msp2*. **A** Frequencies of 3D7 allelic family, **B** frequencies of FC27 allelic family, **C** prevalence of *msp2* repeat polymorphism allelic families of *Plasmodium* *falciparum* in clinical isolates collected in 2015, 2016, and 2021 from Western Gambia. *bp* base pairs

There was a drastic reduction of malaria prevalence in The Gambia from 4% in 2010 to 0.2% in 2017 [26]. However, since 2017 till present the overall countrywide prevalence still stands at 0.2% despite continuous control efforts. This is probably due to a stall or a slight rebound of infections in some parts of the country as indicated by the world malaria reports in recent years [18, 27, 28]. This could affect the genetic diversity of infections and patterns of invasion. Therefore, we genotyped the *P. falciparum* isolates for *msp2* gene repeat polymorphisms to investigate whether parasites collected in the 3 years were genetically different. Alleles were classified according to the size of amplified fragments. Thirty-two individual alleles were detected with 22 alleles for 3D7 (fragment range 230–700 bp) (Fig. 1A) and 10 alleles for FC27 (fragment range 250–450 bp) (Fig. 1B). The frequencies of all alleles detected in the 3D7 family was < 20% and the 350 bp allele was the most prevalent in both 3D7 and FC27 families (18% and 39%, respectively).

The frequency of likely monogenomic infections with single 3D7 allele was higher in 2016 (61.5%) compared to 2015 (40%) and 2021 (40.9%). However, these differences were not statistically significant (*P* = 0.37). Furthermore, there was an increase in polygenomic infections of the same 3D7 allelotype in 2021 (13.6%) compared to 2015 (6.6%) and 2016 (7.7%), but these differences were also not statistically significant (*P* = 0.4). Somewhat similarly, isolates with the FC27 allelotype had a higher frequency (33.3%) of likely monogenomic infections in 2015, while polygenomic FC27 types were detected in 2016 and 2021. Complex (polygenomic) infections with both the 3D7/FC27 allelic types were more prevalent in the 2021 isolates with a frequency of 36.6% compared to 2015 (20%) and 2016 (15.4%) (Fig. 1C).

Overall, the presence of multiple clones in a single infection as defined by the multiplicity of infection (MOI) index was higher in 2021 isolates (1.55) than in 2015 (1.33) and 2016 (1.27). This was contrary to an overall decrease in the heterozygosity (He) of the *msp2* gene from 2015 (0.29) through 2016 (0.2) to 2021 (0.06) (Table 2).

**Table 2:** MOI and heterozygosity of msp2 gene of *Plasmodium falciparum* across 3 years

Variables	2015	2016	2021
MOI	1.33	1.27	1.55
He	0.29	0.2	0.06

*MOI* multiplicity of infection, *He* heterozygosity

### Transcript analysis of invasion ligands

**Fig. 2:**
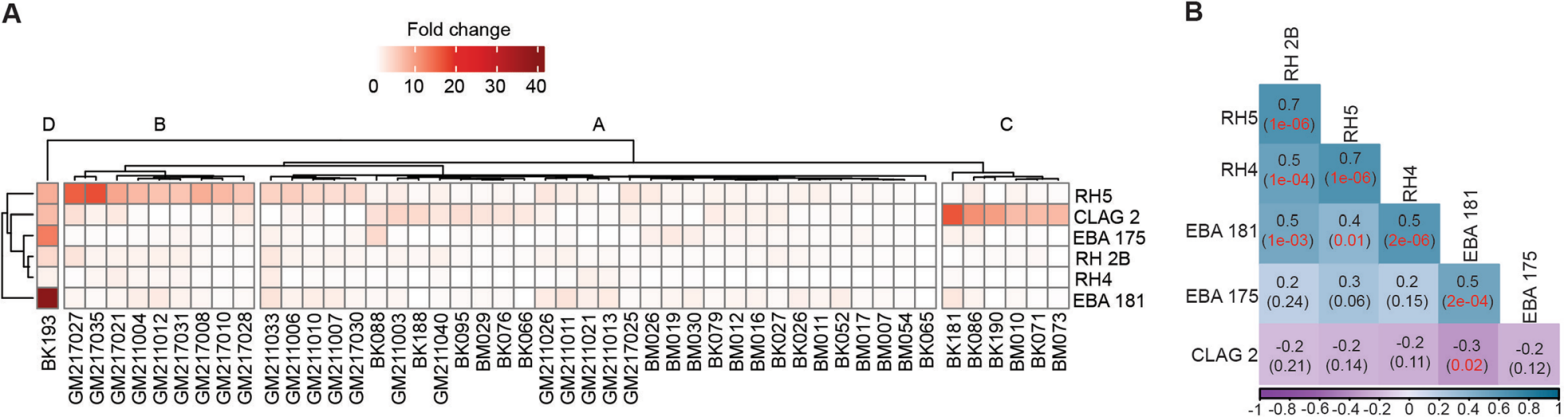
Relative gene expression and correlation between gene expression levels. **A** Hierarchical clustering heatmap of relative expression levels of six *Plasmodium falciparum* ligand genes (rows) between clinical isolates (columns). White to light red colour signifies lower expression while brown colour indicates higher expression. **B** Correlation between gene expression levels; numbers out of the brackets in square boxes represent the correlation coefficients while those in brackets represent the *P* values. Significant *P* values are indicated in red

Specific merozoite ligand genes are predominately expressed by *P. falciparum* isolates during the invasion process and could determine the pathways used. To determine variation in transcript levels of six invasion ligands (CLAG-2, EBA-175, EBA-181, RH-2B, RH4, and RH5), each isolate was cultured ex vivo to predominantly schizont stages and bulk RNA extracted. A total of 48 of the 65 isolates genotyped were successfully cultured for transcript analysis [2015 (*n* = 12), 2016 (*n* = 15), and 2021 (*n* = 21)]. For the other 17 isolates, data were not available due to either poor growth in culture or too low RNA yield after extraction to allow for reliable transcript quantification. The combined transcript profiles across all ligands grouped the isolates into four main clusters (A, B, C, and D) (Fig. 2A). Transcript level profiles for isolates clustered RH4 and RH-2B as well as RH5 and RH-2B together, which are associated with sialic acid-independent invasion pathway, while EBA-181 and EBA-175 genes associated with sialic acid-dependent invasion pathway also clustered together (Fig. 2A). Thus, significant positive correlations were observed between EBA-175 and EBA-181 (Spearman’s *r* = 0.5, *P* = 2e−04), RH-2B and RH4 (*r* = 0.5, *P* = 0.001), and RH5 and RH4 (Spearman’s *r* = 0.7, *P* = 1e−06) (Fig. 2B).

Overall, CLAG2 (mean 2.61) and RH5 (mean 2.93) showed the relative highest copy numbers of transcripts across the 3 years with no significant temporal differences between their expression (*P* = 0.41), followed by EBA-181 (mean 1.05) and EBA-175 (mean 1.03) (Additional file 1: Fig. S1). In addition, analysis of the invasion efficiency of isolates according to the ligand genes expression clusters in Fig. 2A showed no statistically significant differences in invasion efficiency of all enzyme treatments across all clusters (Additional file 1: Fig. S2).

**Fig. 3:**
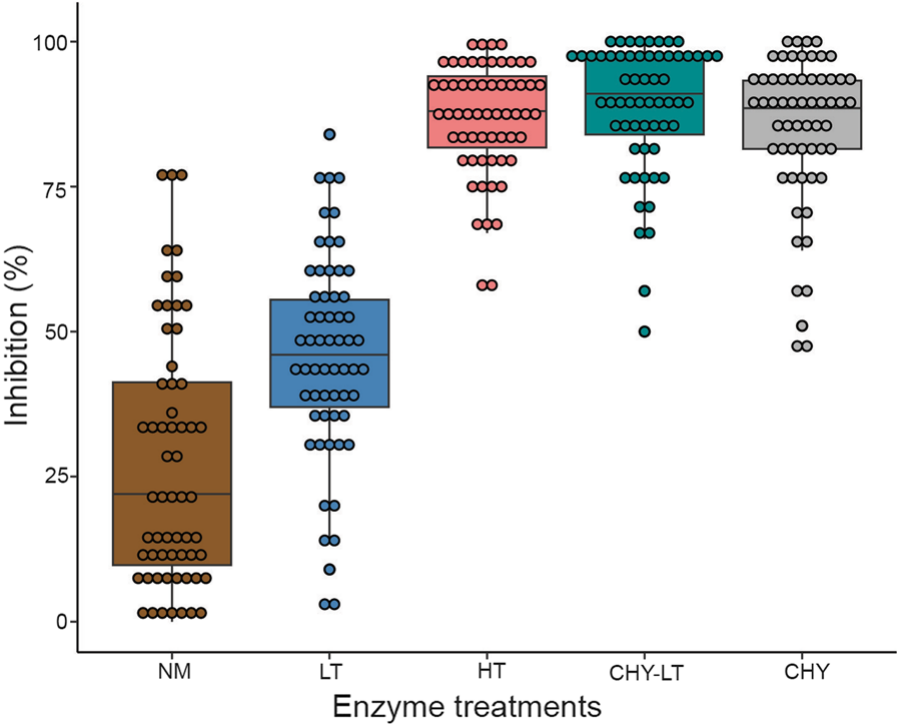
Invasion phenotypes of *P. falciparum* isolates across 3 years in The Gambia. *NM* neuraminidase, *LT* low trypsin, *HT* high trypsin, *CHY_LT* chymotrypsin and low trypsin, and *CHY* chymotrypsin. Each point represents an isolate while the vertical line of each box represents the median.* P* < 0.05 was considered statistically significant

### Invasion inhibition of *P. falciparum* clinical isolates across three years

Following depletion of receptors using various enzymes, we observed very low levels (mean 27.3%) of invasion inhibition into neuraminidase (NM)-treated erythrocytes by isolates from all 3 years. Higher levels of inhibition were observed for low trypsin (LT; 45.6%, *P* = 6.1 e−6), high trypsin (HT; 86.6%, *P* = 2 e−16), and chymotrypsin (CHY; 84.9%, *P* = 2 e−16). Combining chymotrypsin and trypsin (CHY_LT) resulted in higher invasion inhibition (mean 88.9%) than when the enzymes were used separately but these differences were not statistically significant (CHY_LT and CHY *P* = 0.058, CHY_LT and HT *P* = 0.065 (Fig. 3).

**Fig. 4:**
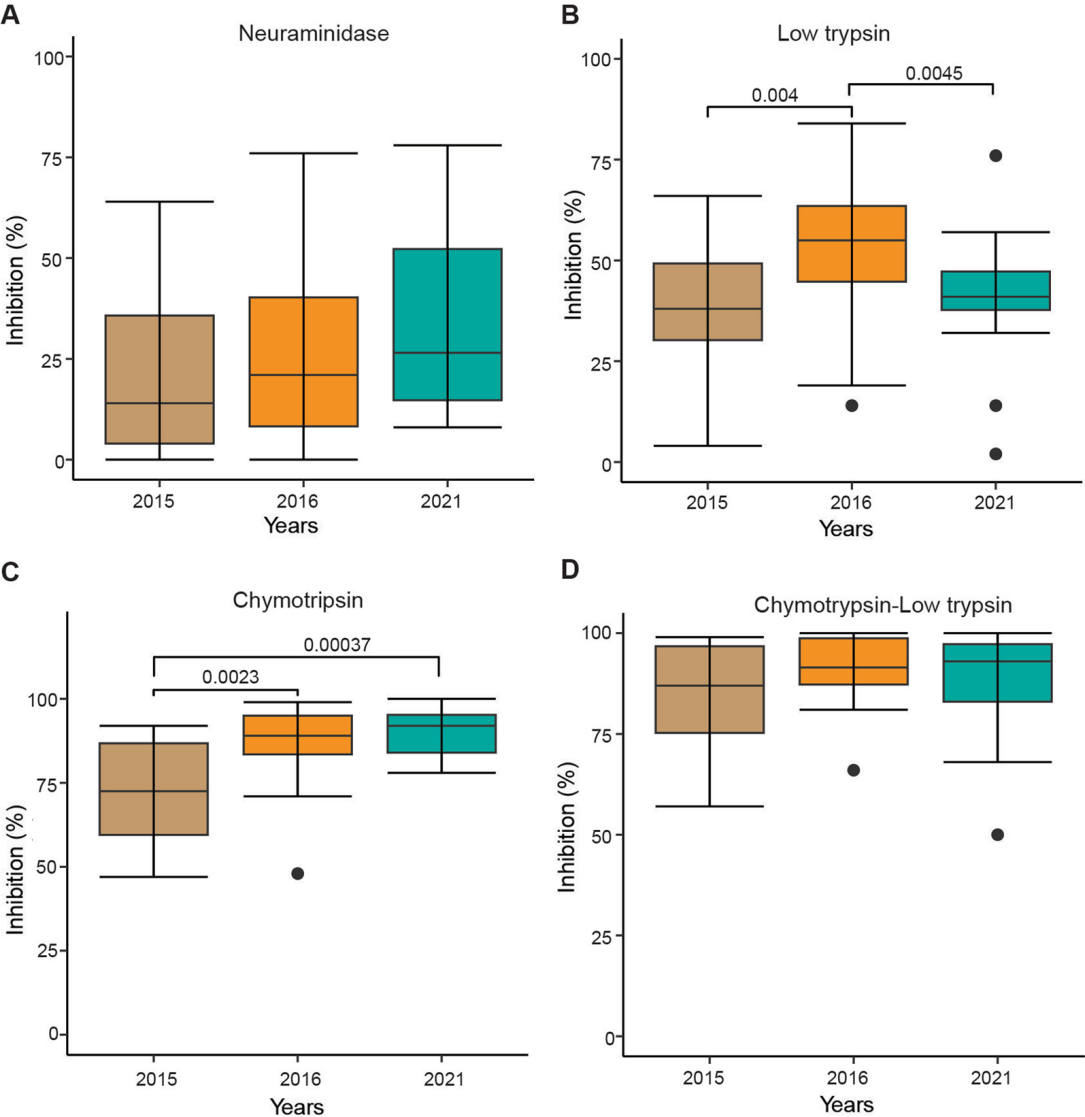
Comparison of erythrocyte invasion inhibition phenotypes of *Plasmodium falciparum* isolates across 3 years in The Gambia. **A** Neuraminidase, **B** low trypsin, **C** chymotrypsin, and **D** chymotrypsin and low trypsin. Each box shows the invasion efficiencies in enzyme-treated erythrocytes relative to untreated erythrocytes, and the horizontal black lines in each box represent the median. *P* values < 0.05 were considered statistically significant

Comparison of invasion phenotypes across the 3 years showed a trend towards increased neuraminidase inhibition between 2015 to 2021 (mean: 2015 = 22.21%, 2016 = 26.69%, and 2021 = 31.6%) but the differences were not statistically significant (Kruskal-Wallis test, *P* = 0.3) (Fig. 4A). Low trypsin-treated erythrocytes resulted in a significant increase (*P* = 0.004 between 2015 and 2016 and 0.0045 between 2016 and 2021) in invasion inhibition between years with a mean of 37% in 2015 and 41.1% in 2021, and isolates collected in 2016 showed a higher (mean 53.65%) inhibition rate compared to the preceding year 2015 isolates (uncorrected *P* = 0.004, *P* corrected for multiple pairwise comparisons = 0.012) (Fig. 4B). Furthermore, inhibition by chymotrypsin was significantly higher in 2016 isolates (mean 87.38%) compared to 2015 (Mann-Whitney uncorrected *P* = 0.0023, corrected *P* = 0.007 (Fig. 4C) and even more in 2021 (mean 90.6%) compared to 2015 isolates (Mann-Whitney uncorrected *P* = 0.00037, corrected *P* = 0.001). However, inhibition by this enzyme combination (chymotrypsin and low trypsin) was more effective across all years (mean: 2015 = 84.57%, 2016 = 91.65%, and 2021 = 88.35) (Fig. 4D).

Following the observed invasion inhibition phenotypes and variations across the years, we tested whether the phenotypes also varied by *msp2* allelic families. For the most dominant allelic type (350 bp) for both families (3D7 and FC27), invasion inhibition was significantly higher in 3D7 allelic family only in the case of erythrocyte treatment with high trypsin (*P* = 0.027) (Additional file 1: Fig. S3). For neuraminidase-treated cells the 3D7 type isolates were also less inhibited, even though the difference with FC27 type isolates was not significant.

### Correlation between invasion inhibition by enzyme treatments and age

**Fig. 5:**
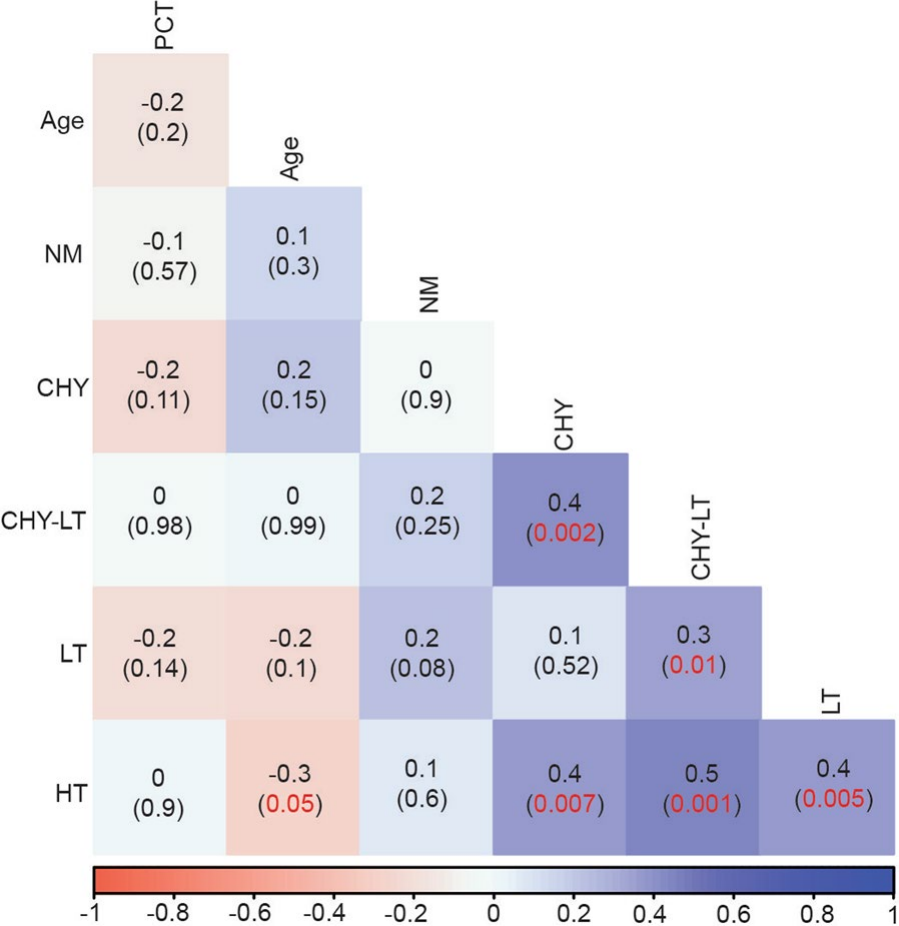
Correlation among enzyme inhibition, age, and parasitaemia. Numbers out of the brackets represent the correlation coefficients while those in brackets indicate the *P*-values with significant *P*-values highlighted in red. *PCT* parasitaemia

The magnitude and pattern of inhibition of invasion by CHY, CHY_LT, and NM were positively correlated with age but negatively correlated with parasitaemia while HT was negatively correlated with age but positively correlated with parasitaemia. These correlations among age, parasitaemia, and enzyme treatments were not statistically significant, although marginally significant for HT inhibition and age (0.05) (Fig. 5). Further grouping of the study participants into three age groups as young children (0–5 years), older children (6–17 years), and adults (18 years and above) resulted in significant differences among age groups and enzyme treatments (Additional file 1: Fig. S4). Inhibition by low trypsin and chymotrypsin significantly correlated negatively with parasitaemia in younger children (Spearman’s r = − 0.76 and r = − 0.82, respectively) (Additional file 1: Fig. S4).

## Discussion

This study characterized and compared the genetic diversity, ligand genes expression, and invasion phenotypes of *P. falciparum* clinical isolates across 3 years in Western Gambia, where enhanced control interventions for over a decade have led to a significant decline in malaria transmission and clinical incidence. This overall decline in malaria transmission was reflected by lower heterozygosity in *msp2* polymorphic repeat alleles in this study compared to previous reports. However, world malaria reports in the recent years covered by the study indicate a slight rebound of infections in The Gambia [18, 27, 28]. Thus, we observed an increase in polygenomic infections in the infections sampled from 2021 compared to 2015 and 2016, despite the lower overall heterozygosity. There were more infections in 2021 with more than a single clone despite the fewer overall number of clones in circulation. This could be attributed to the slight increase in transmission. Such a phenomenon had been described between populations in Cameroon, where high multiplicity of infections was observed despite the low number of circulating genotypes [29]. This observation is unexpected since The Gambia is in the pre-elimination phase of malaria and MOI is expected to be decreasing over the years. Moreover, it is generally expected that an increase in MOI should lead to increasing heterozygosity but that was not the case in this study [30–32]. It is probable that despite the low transmission in the Western region of The Gambia, multiple clones with few polymorphic loci have been maintained or are circulating in the population or imported in this most urban region of the country. These results should be interpreted with caution because of the small sample size, and sampling only from Western Gambia does not reflect the entire country. Moreover, only the *msp2* gene was genotyped, and the pattern could differ if diversity at other polymorphic loci such as microsatellites and genome-wide single-nucleotide polymorphisms (SNPs) was evaluated.

Parasitaemia is established in malaria following several merozoite ligand-erythrocyte receptor interactions, merozoite invasion and replication in erythrocytes [10]. Erythrocyte invasion can occur through several alternative pathways, which could be determined by the expression levels and type or use of different ligand combinations [33–35]. Analyses of transcript levels relative to apical membrane antigen 1 (AMA1) gene for selected invasion ligands in our clinical isolates showed significant differences in the rhoptry protein, RH5 [36, 37], relative to EBA-181 and EBA-175. RH5 is a host tropism determinant and an unavoidable ligand in the invasion process, binding to basigin (CD147) and P113 [38, 39]. It is one of the leading blood-stage *P. falciparum* malaria vaccine antigens, and its higher expression relative to EBA-175 is in contrast to previous studies in Ghana and The Gambia, where dominance of EBA-175 transcripts and SA-dependent pathways was reported [17, 40]. Unlike isolates from these previous years, most of those we characterised here were SA independent. RH5 expression correlated strongly with that of RH4 and RH2b, two ligands also important for SA-independent invasion, supporting the switch from SA-dependent invasion in The Gambia. Parasites may vary invasion pathways to non-EBA-dependent and SA-independent pathways, given immune pressure against the EBA family of proteins as demonstrated from disruption of EBA-175 gene [41]. Malaria has been in a decline in The Gambia, with expected reduction in population level immunity [42, 43]. While this should ideally reduce the pressure on EBA-175, and allow for sustained SA-dependent invasion, the reverse seen here points to immune pressure not being the only factor in pathway switch. Indeed, pathway switch to more efficient mechanisms may be conditioned by the environment as seen during in vitro culture adaptation [44]. High expression was also detected for CLAG-2, a gene that is associated with the rhoptry bulb, and a member of a multigene family of rhoptry proteins, implicated in cytoadherence, infected cell permeability, and invasion [45, 46]. These results together further indicate that the level of antigen expression alone cannot explain the variation in patterns of invasion pathways, as these correlate with other infection matrices. For example, there was a positive correlation between RH5 expression and parasitaemia as previously reported from The Gambia [17], Ghana [40], Mali [47], and Papua New Guinea [48]. RH5 is an essential invasion antigen, a potent vaccine candidate in advanced development. Its protective role against infections and disease needs to be further evaluated as malaria parasite populations evolve against intense interventions with changing epidemiology.

The increased use of SA-independent receptors and neuraminidase resistance from 2015 to 2021 in the Western region of The Gambia may be because of adaptation to reduced transmission pressure. Clinical isolates of *P. falciparum* from other regions in Africa have also shown similar levels of resistance to neuraminidase treatment [23, 40, 49]. Most of these were also isolates from recent populations, in contrast to previous findings of field isolates collected over a decade ago in The Gambia, where the parasites’ ability to invade neuraminidase-treated erythrocytes was much higher [16, 17]. Malaria transmission during the earlier studies was more intense in Western Gambia, and the variation observed here could also be due to some of the precious specimens tested from severe malaria cases compared to the mostly low parasitaemia uncomplicated cases in this study [17]. There is also the possibility of variations in the activities of commercial neuraminidase enzyme batches used. Overall, the use of different invasion pathways remains heterogeneous, and the molecular patterns determining this remain complicated, needing further evaluation as parasite ligands are targeted for vaccine development. This study is limited by the small number of specimens from each year, a factor in other invasion studies most likely due to the significant capacity and resources needed for parasite culturing and cellular assays.

## Conclusion

In conclusion, this study showed a small recent increase in low complexity polygenomic infections in the Western region of The Gambia and suggests that recent clinical isolates of *P. falciparum* from uncomplicated malaria are increasingly using sialic acid-independent invasion pathways against the sialic acid-dependent alternative. With The Gambia accelerating interventions towards pre-elimination, and possibility of new elimination tools (e.g., vaccines), continuous molecular and phenotypic surveillance of the parasite population is necessary.

### Supplementary Information

**Additional file 1: Figure S1.** Fold gene expression of genes encoding six invasion ligands in ex vivo schizont-stage cultures of *Plasmodium falciparum* clinical isolates across 3 years in The Gambia. (A) combined expression of isolates across all years (*n* = 48), (B) 2015 isolates (*n* = 12), (C) 2016 isolates (*n* = 15), and (D) 2021 isolates (*n* = 11). Horizontal lines indicate the mean for each transcript across all isolates sampled in each year, and each dot denotes the transcript for each gene in a single clinical isolate. **Figure S2.** Invasion inhibition phenotypes of *Plasmodium falciparum* clinical isolates according to gene expression clusters. **Figure S3.** Invasion inhibition phenotypes of *Plasmodium falciparum* clinical isolates by dominant allele per *msp2* gene allelic family. Neuraminidase (NM), low trypsin (LT), high trypsin (HT), chymotrypsin and low trypsin (CHY_LT), and chymotrypsin (CHY). **Figure S4.** Scatter matrix plot of enzyme treatment and parasitaemia (x-axes and y-axes) between different age groups. Within each panel, each bullet point represents the % invasion inhibition of each enzyme. The asterisk (*) represents groups with significant *P*-values. NM = neuraminidase, LT = low trypsin, HT = high trypsin, CHY_LT = chymotrypsin/low trypsin, PCT = parasitaemia, and CHY = chymotrypsin.

## Data Availability

Data supporting the conclusion of this article are included within the article.

## Abbreviations

Rh5: Reticulocyte binding protein homolog 5
Rh4: Reticulocyte binding protein homolog 4
Rh2b: Reticulocyte binding protein homolog 2b
CR1: Complement receptor 1
CLAG-2: Cytoadherence linked asexual protein 2
EBA-181: Erythrocyte binding antigen 181
EBA-175: Erythrocyte binding antigen 175
Gly: Glycophorin
RDT: Rapid diagnostic test
EDTA: Ethylenediaminetetraacetic acid
SNPs: Single nucleotide polymorphisms
AMA1: Apical membrane antigen 1
CTFR: Cell trace far red
SA: Sialic acid
