# Supplementary material for: Recent increase in low complexity polygenomic infections and sialic acid-independent invasion pathways in Plasmodium falciparum from Western Gambia
Source: Parasit Vectors. 2023 Aug 31;16:309. doi: 10.1186/s13071-023-05929-4 (PMC10472613; doi:10.1186/s13071-023-05929-4)
Supplement: Supplementary file 1 — Additional file 1: Figure S1. Fold gene expression of genes encoding six invasion ligands in ex vivo schizont-stage cultures of Plasmodium falciparum clinical isolates across 3 years in The Gambia. (A) combined expression of isolates across all years (n = 48), (B) 2015 isolates (n = 12), (C) 2016 isolates (n = 15), and (D) 2021 isolates (n = 11). Horizontal lines indicate the mean for each transcript across all isolates sampled in each year, and each dot denotes the transcript for each gene in a single clinical isolate. Figure S2. Invasion inhibition phenotypes of Plasmodium falciparum clinical isolates according to gene expression clusters. Figure S3. Invasion inhibition phenotypes of Plasmodium falciparum clinical isolates by dominant allele per msp2 gene allelic family. Neuraminidase (NM), low trypsin (LT), high trypsin (HT), chymotrypsin and low trypsin (CHY_LT), and chymotrypsin (CHY). Figure S4. Scatter matrix plot of enzyme treatment and parasitaemia (x-axes and y-axes) between different age groups. Within each panel, each bullet point represents the % invasion inhibition of each enzyme. The asterisk (*) represents groups with significant P-values. NM = neuraminidase, LT = low trypsin, HT = high trypsin, CHY_LT = chymotrypsin/low trypsin, PCT = parasitaemia, and CHY = chymotrypsin. [file 13071_2023_5929_MOESM1_ESM.doc]

**Recent increase in low complexity polygenomic infections and sialic acid-independent invasion pathways in *Plasmodium falciparum* from Western Gambia**

**Nora Nghochuzie Nganyewo^1,2^, Fatoumata Bojang^1^, Eniyou Cheryll Oriero^1^, Ndey Fatou Drammeh^1^, Ajibola Olumide^1^, Haddijatou Mbye^1^, Aminata Seedy Jawara^1^, Simon Corea^1^, Gordon Akanzuwine Awandare^2^, Umberto D’Alessandro^1^, Lucas N. Amenga-Etego^2^, and Alfred Amambua-Ngwa^1*^**

^1^Medical Research Council Unit The Gambia at London School of Hygiene and Tropical Medicine, Banjul, The Gambia.

^2^West African Centre for Cell Biology of Infectious Pathogens (WACCBIP), University of Ghana, Accra, Ghana.

*Correspondence: alfred.ngwa@lshtm.ac.uk, [angwa@mrc.gm](mailto:angwa@mrc.gm)

**Results**

**Fold expression of invasion ligand genes of *P. falciparum* clinical isolates across 3 years**

CLAG2 (mean 2.61) and RH5 (mean 2.93) were the most abundant genes expressed with no significant difference between their expression (*P* = 0.41), followed by EBA-181 (mean 1.05) and EBA-175 (mean 1.03). RH4 was the least expressed with a mean of 0.43 (Figure. S1A). When compared across the 3 years, the expression of CLAG2 was higher in parasites collected in 2015 than RH5 (*P* = 0.007) (Figure. S1B) unlike in 2016 where there was no significant difference between CLAG2 and RH5 (*P* = 0.55) (Figure. S1C). Moreover, RH5 was more highly expressed in 2021 isolates than CLAG2 (*P* < 0.001) (Figure. S1D).


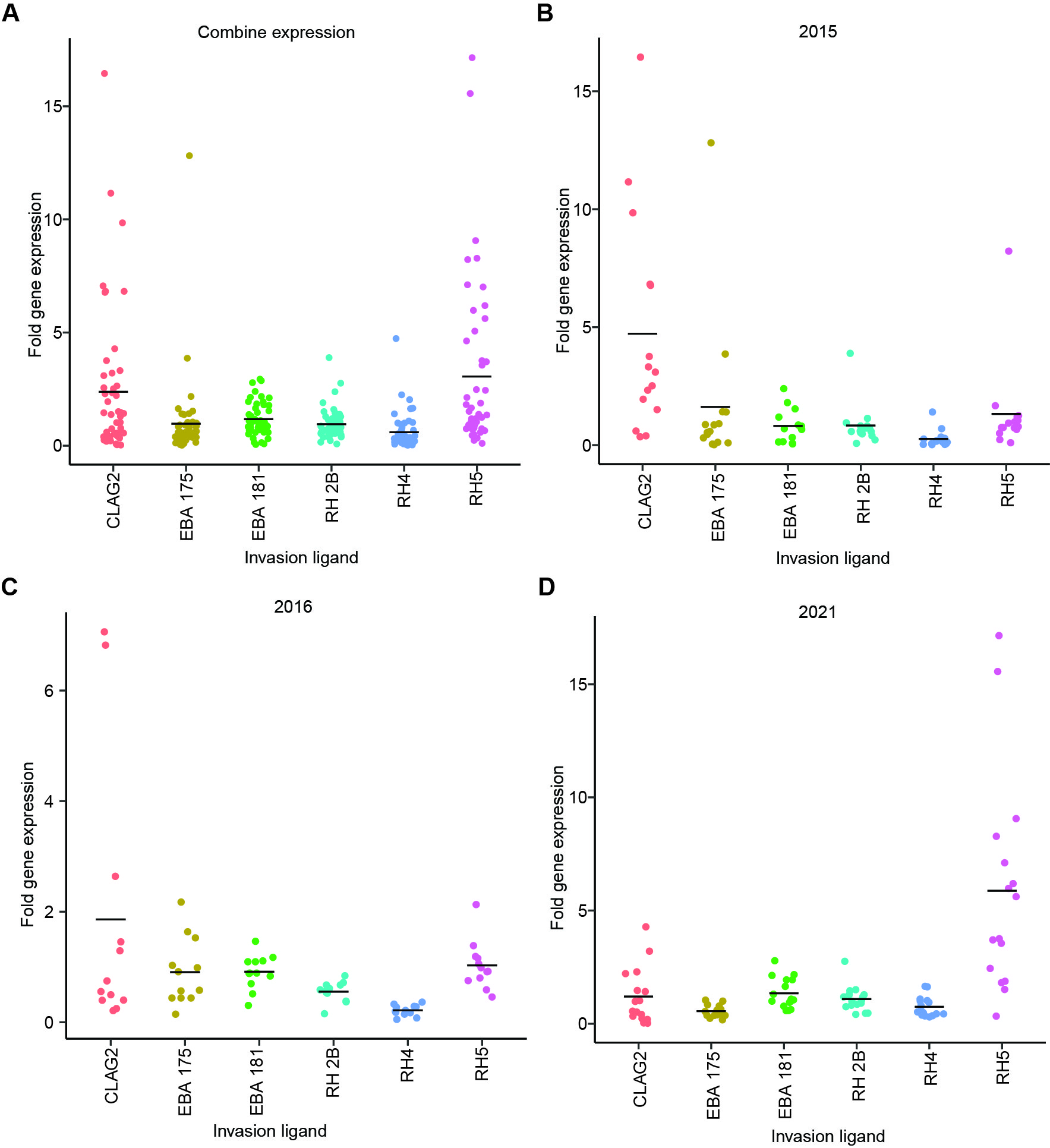


**Figure. S1:** Fold gene expression of genes encoding 6 invasion ligands in *ex vivo* schizont-stage cultures of *P. falciparum* clinical isolates across 3 years in The Gambia. (A) combined expression of isolates across all years (n= 43), (B) 2015 isolates (n=12), (C) 2016 isolates (n=15), and (D) 2021 isolates (n= 16). Black horizontal lines indicate the mean for each transcript across all isolates sampled in each year and each dot denotes the transcript for each gene in a single clinical isolate.

**Invasion efficiency by ligand genes expression clusters**

Further analysis of the invasion efficiency of isolates according to the ligand genes expression clusters in figure 2A showed no statistically significant differences in invasion efficiency across all clusters within enzyme treatments (Kruskal-Wallis test, *P* = 0.46, 0.89, 0.23, 0.95, and 0.32 for NM, LT, HT, CHY_LT, and CHY respectively) (Figure. S2).


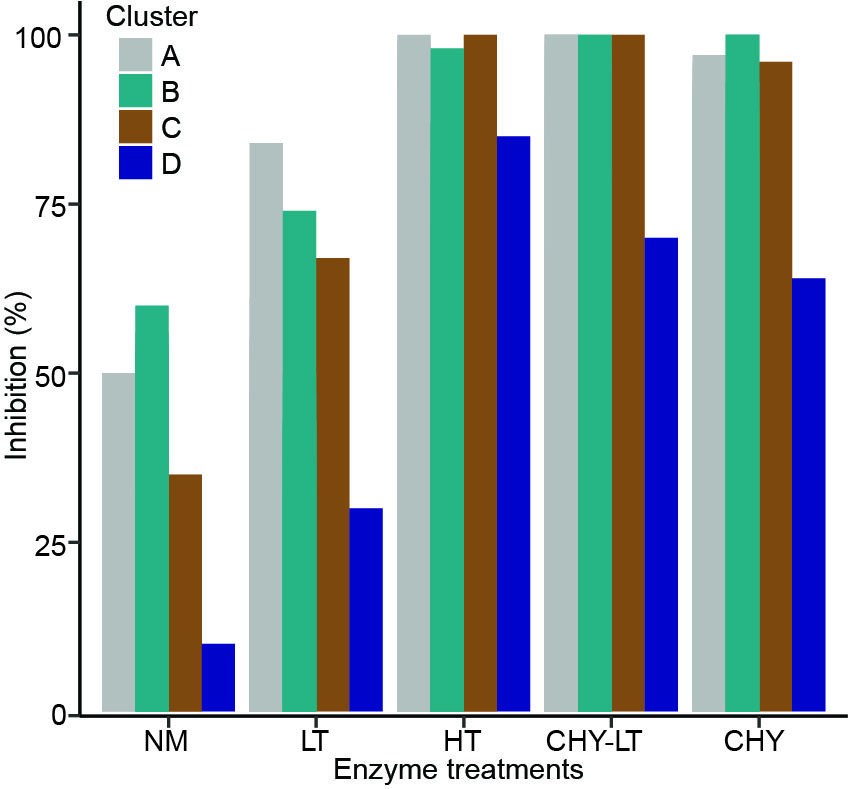


**Figure. S2:** Invasion inhibition phenotypes of *P. falciparum* clinical isolates according to gene expression clusters.

**Invasion inhibition patterns of isolates from different *msp2* allelic families**

Analysis of *msp2* gene by allelic families resulted in varying inhibition efficiency across all enzyme combinations, but these differences were not statistically significant (Kruskal- Wallis *P* = 0.92, 0.3, 0.07, 0.7, and 0.57 for NM, LT, HT, CHY_LT, and CHY respectively). Moreover, although both allelic families had the same dominant allele (350bp), further analysis of invasion inhibition by the dominant alleles per allelic group identified statistically significant differences between the two group with high trypsin treatment only (*P* = 0.027) (Figure. S3).


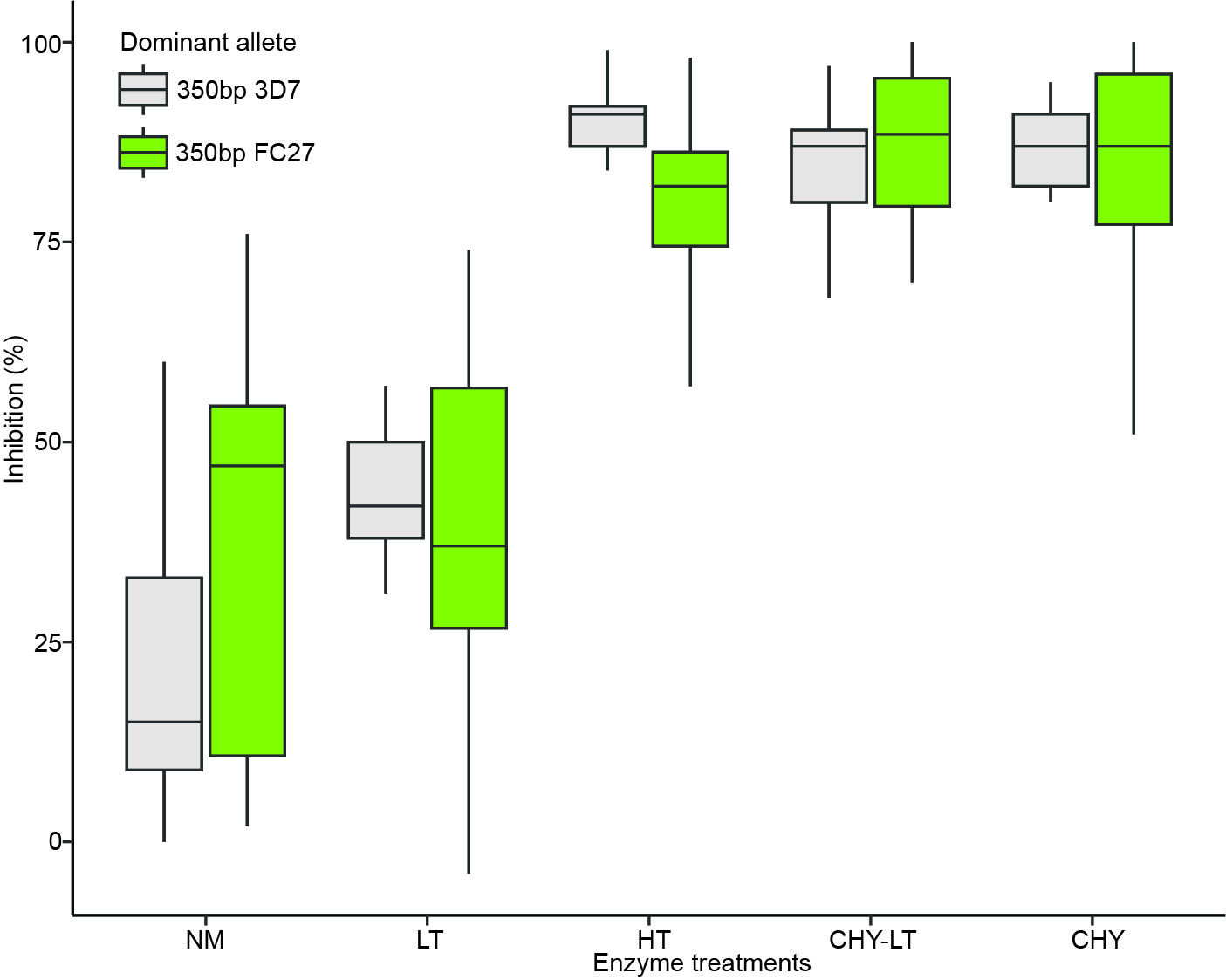


**Figure. S3:** Invasion inhibition phenotypes of *P. falciparum* clinical isolates by dominant allele per *msp2* gene allelic family. Neuraminidase (NM), low trypsin (LT), high trypsin (HT), chymotrypsin and low trypsin (CHY_LT), and chymotrypsin (CHY). bp = base pairs.

**Correlation between enzyme treatments, age groups and parasitaemia**

Given that 2015 and 2016 isolates were collected from children < 14 years while 2021 isolates were collected from all age groups (both children and adults), we sought to ask if different ages and parasitaemia might have contributed to the observed differences apart from the years of sample collection. To this effect, we grouped the study participants into different age groups as follows: 0-5 years as young children, 6-17 years as older children, and 18 years and above as adults. We then went further to do a multiple-variable correlation analysis incorporating the different age groups, parasitaemia, and the different enzyme treatments. Significantly negative correlations were observed between parasitaemia (PCT) and LT treatment (*r* = -0.755, *P* = 0.045) among young children while significantly positive correlations were observed between NM and LT treatments (*r* = 0.434, *P* =0.033), CHY and CHY_LT treatments (r = 0.495, P = 0.008) among older children (Figure S4). All statistically significant correlations between enzyme treatments or between parasitaemia and enzyme treatments were either among young children or older children.


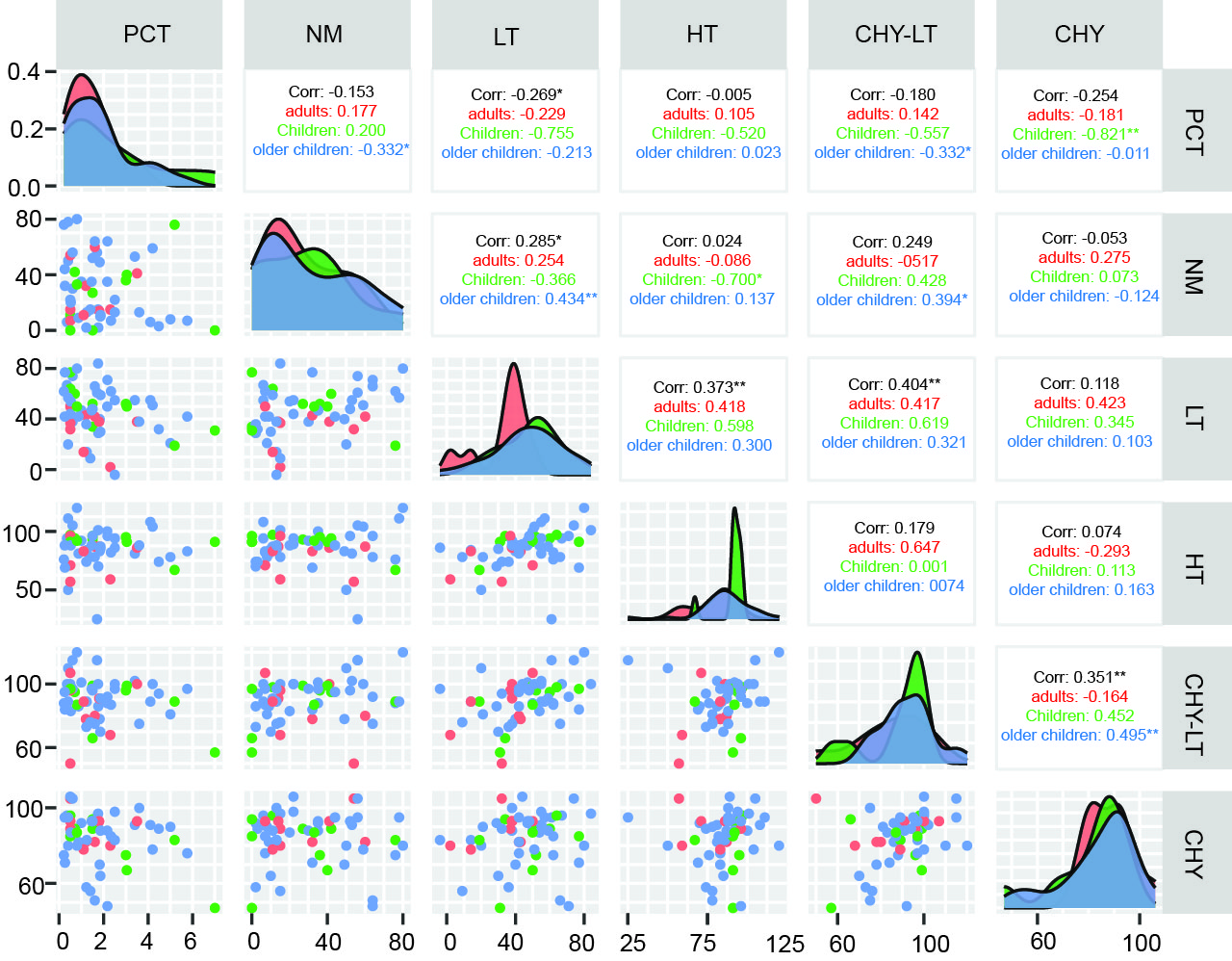


**Figure. S4:** Scatter matrix plot of enzyme treatment and parasitaemia (x-axes and y-axes) between different age groups. Within each panel, each bullet point represents the invasion inhibition (%) of each enzyme. The asterisk (*) represents groups with significant *P*-values, NM = neuraminidase, LT = low trypsin, HT = high trypsin, CHY_LT = chymotrypsin/low trypsin, PCT = parasitaemia, and CHY = chymotrypsin.
